# TMT-Based Proteomic Analysis of Human Spermatozoa from Unexplained Recurrent Miscarriage Patients before and after Oral Antioxidant Treatment

**DOI:** 10.3390/biomedicines10082014

**Published:** 2022-08-19

**Authors:** Alba Fernandez-Encinas, Jordi Ribas-Maynou, Agustín García-Peiró, Sergio Garcia-Segura, Olga Martinez-Pasarell, Joaquima Navarro, Maria Oliver-Bonet, Jordi Benet

**Affiliations:** 1 Departament de Biologia Cel·lular, Fisiologia i Immunologia, Universitat Autònoma de Barcelona, 08193 Barcelona, Spain; 2 Centro de Infertilidad Masculina y Análisis de Barcelona (CIMAB), Sant Quirze del Vallès, 08193 Barcelona, Spain; 3 Fundació Puigvert Hospital de Sant Pau i de la Santa Creu, 08193 Barcelona, Spain

**Keywords:** sperm, recurrent miscarriage, antioxidants, sperm DNA fragmentation, proteomics, male infertility

## Abstract

Recently, sperm quality and the presence of double-stranded breaks (DSB) has been pointed out as a possible cause of recurrent miscarriage, and the use of antioxidants has expanded as a treatment for male infertility. The aim of the present study was to analyze the proteomic effects of antioxidants on sperm from RM patients with high incidence of DSB. Proteomic analysis was performed using a tandem mass tag labeling technique, and subsequently compared with the PANTHER database for DEPs, and the STRING database for protein–protein interactions (PPI). Differentially expressed proteins (DEPs) both before and after antioxidant oral treatment were identified. PPI involving DEPs clustered into networks related to cell metabolism, cytoskeleton, and DNA damage. Results show that the sperm proteomic profiles before and after antioxidant treatment do not significantly differ from each other. However, some DEPs found after the antioxidant treatment shifted towards a DEPs profile typical of fertile donors. This indirect measurement suggests an improvement caused by antioxidants on the expression of several proteins. Among them were proteins involved in sperm DNA remodeling (LMO7, MMP28, BNC2, H2B, and PRDM2). The results presented here represent the first approach in the analysis and repair of the proteomic change caused by antioxidants in recurrent miscarriage patients, elucidating biomarkers that may be useful for the diagnosis and further sperm selection in this type of patient. Further studies should be conducted to validate the usefulness of these biomarkers in larger study groups.

## 1. Introduction

Pregnancy loss affects nearly 50% of embryos [1,2]. It may happen before the embryo is successfully implanted in the uterus, between embryo implantation and the recognition of biochemical pregnancy, or after pregnancy is clinically detected through confirming a heartbeat. In the first two situations, pregnancy loss cannot clinically be differentiated from infertility, but in the third case, pregnancy loss results in miscarriage. Miscarriage has a reported incidence of about 15%, and between 25% and 50% of pregnant women will experience one or more during their reproductive life [2]. The term recurrent miscarriage (RM) refers to two or more consecutive clinical pregnancy losses that happen before reaching the end of the first trimester or early in the second trimester. It affects about 1% to 5% of the couples trying to conceive [3,4]. Several causes have been linked to RM, the most prevalent (50–60%) being cytogenetic abnormalities such as trisomy, polyploidy, and monosomy [5,6,7,8]. However, it has also been associated with structural anomalies of the uterus, infections, endocrine abnormalities, immune dysfunctions, antiphospholipid syndrome, and thrombophilic disorders [2,9,10].

Both sperm and oocyte defects can cause infertility. Different studies have shown that sperm DNA integrity is essential to embryo quality, and that DNA damage is associated with male-factor infertility [11,12]. More specifically, two different alterations of sperm chromatin integrity have been associated with a negative impact on pregnancy: on one hand, single-stranded breaks (SSBs) have been related to a lack of pregnancy [13,14,15,16], on the other hand, the presence of double-stranded DNA breaks (DSBs) might be responsible for a higher miscarriage risk [17]. The presence of SSBs has been associated with an increase in reactive oxygen species (ROS) and/or decreased overall antioxidant protection [18,19], for which the assessment of seminal plasma is also important [20]. To counteract the detrimental effects of ROS, oral antioxidant treatments are routinely applied to infertile males, despite the fact that the Cochrane report on antioxidants for male subfertility shows no conclusive results supporting their use [21]. Regarding DSBs, it has been shown that an increased presence of DNA breaks at the sperm toroid linker regions (TLR) is associated with a higher risk of recurrent pregnancy loss [17] and a higher risk of implantation failures in couples undergoing intracytoplasmic sperm injection (ICSI) [22]. These specific DSBs have been shown to remain associated with the sperm nuclear matrix until fertilization, thus supporting the possibility that they are repaired during the first stages of embryo development. If this is not the case, they have been shown to cause genetic defects such as loss of chromosomal fragments or genetic alterations [17].

Despite the advances in the search for male-factor-related biomarkers for RM, such as the presence of DSB, new molecular biomarkers need to be identified in order to better understand the physiopathology of this disease. In this sense, a study by Xue et al. [23] identified 38 proteins that were significantly altered in RM patients, mostly related to metabolic processes, biological regulation, and protein binding activities [23]. Additionally, a study by Sutovsky et al. [24] identified that patients with high SPTRX3 protein content in semen are prone to RM [24]. Finally, other authors showed an association between RM and upregulation of prostate-specific antigen or clusterin in seminal plasma and the downregulation of annexin A3 [25].

Since the application of high-throughput proteomic approaches may help the search for new biomarkers specifically related to human disease, the aim of the present work was to identify differences in the expression of sperm proteins in patients with recurrent miscarriage who present increased incidence of double-stranded DNA breaks. Additionally, we aimed to assess whether an antioxidant treatment caused a change in the differentially expressed proteins. 

## 2. Materials and Methods

### 2.1. Samples and Study Design

Semen samples were collected from five fertile donors and five infertile patients with RM, both before and after oral antioxidant treatment. The main inclusion criterium for the semen samples obtained from RM patients was the presence of double-stranded sperm DNA fragmentation. After conducting a comet assay to test for the inclusion criteria, samples were classified into three groups according to their clinical features, and double-stranded DNA damage was measured using a comet assay. In the first group, fertile donors (FD) included five fertile donors that had achieved a clinical pregnancy during the last year. All donors presented normal semen parameters according to WHO 2010 (volume > 1.5 mL, sperm concentration > 15·× 10^6^ sperm/mL, progressive motility > 32%, and normal morphology using Kruger criteria > 4%), alkaline comet assay (<45%), and neutral comet assay (<60%). Additionally, fertile donors did not show any detectable infection in semen and presented a normal karyotype. The second group, the recurrent miscarriage (RM) group, included samples from five RM patients with normal semen parameters according to WHO 2010, low alkaline comet assay (<45%), and high neutral comet assay (>60%). Furthermore, patients presented a normal karyotype and did not depict any detectable infection in semen. In addition, for the RM group, couples did not present any of the most common female factors related to RM (advanced maternal age, karyotype, antiphospholipid antibodies, uterine abnormalities, and thrombophilia). The third sample group comprised the same RM patients (group 2) after receiving oral antioxidant treatment (RM-AO) for three months. This treatment consisted of 1500 mg L-carnitine, 20 mg coenzyme Q10, 60 mg vitamin C, 10 mg vitamin E, 200 µg folic acid, 1 µg vitamin B12, 50 µg selenium, and 10 mg zinc. This formula has already been used in other trials, showing its usefulness in improving sperm quality and global DNA damage [26,27].

All samples were collected by masturbation after 2–5 days of recommended abstinence. Written informed consent was obtained for all patients, and the Parc Taulí Hospital ethics committee approved the present study with registration number 2017902.

#### 2.1.1. Semen Collection and Cryopreservation

After liquefaction, routine semen analysis was performed according to WHO (2010), and the semen samples were subsequently divided into two aliquots; 500 µL of each unprocessed semen sample was cryopreserved with TEST-yolk buffer (14% glycerol, 30% egg yolk, 1.98% glucose, 1.72% sodium citrate) and stored at −80 °C until use in TMT analysis or comet assay.

#### 2.1.2. DNA Integrity Tests: Alkaline and Neutral Comet Assay

Comet assays were performed via two different variants, alkaline and neutral, to detect different types of DNA breaks. The complete protocol has been described before by our research group [22]. Briefly, samples were thawed for 30 s at 37 °C and washed in PBS. After adjusting sperm concentration to 1 × 10^6^ sperm/mL, and mixing samples with 1% low-melting-point agarose in a 1:2 proportion, microgels were mounted by pouring 15 μL into two different slides, covering them with a coverslip, and leaving them to cool at 4 °C for 5 min. Then, coverslips were removed, two sperm lysis steps were conducted, and electrophoresis was performed at alkaline pH (pH > 13) for the alkaline comet assay and at neutral pH (pH = 8) for the neutral comet assay. Finally, slides were neutralized, dehydrated, and stained with DAPI (Slowfade Gold antifade, Thermo Fisher Scientific, Whaltham, MA, USA). Evaluation was conducted by classifying 400 sperm as normal or fragmented, following the previously reported criteria [28]. The percentage of fragmented sperm cells was recorded. Statistical differences among groups (Table 1) were assessed using the Mann–Whitney U test for unpaired samples, and using the Wilcoxon test for paired samples.

### 2.2. Proteomic Analysis of Spermatozoa

#### 2.2.1. Sample Preparation for Proteomic Analysis

Each sample was thawed at 37 °C for 30 s and washed three times with PBS to keep sperm cells in pellet form. Sperm cells were lysed with a buffer (50 mM Tris-HCl, 2% SDS, 10 mM dithiothreitol (DTT)), solubilized, and sonicated three times on ice. The lysed cells were centrifuged at 16,100× *g* for 15 min at 4 °C, and supernatant was carefully collected. Proteins were quantified using a Pierce BC protein assay (Thermo Fisher Scientific, Whaltham, MA, USA) following the manufacturer’s protocol.

Finally, proteins were reduced in 5 mM dithiothreitol at 60 °C for 30 min, and subsequently alkylated with 0.5 M iodoacetamide for 30 min in the dark. Proteins were precipitated with cold 100% acetone for 15 min at –20 °C. After centrifugation (16,100× *g*, 4 °C, 15 min), protein pellets were resuspended in 50 mM TEAB (tetraethylammonium bicarbonate) up to a concentration of 1 μL protein/μL solution.

#### 2.2.2. Trypsin Digestion and TMT Labeling

Proteins were digested with trypsin, in a 1:50 mass ratio of trypsin to protein, overnight at 37 °C with constant shaking. Internal controls were established exhibiting the same final sample concentration. Peptides from samples were desalted using a Strata X C18 SPE column (Phenomenex, Torrance, CA, USA) and vacuum-dried. Peptides were labeled with TMT isobaric tags using a 10-plex TMT kit (Thermo Fisher Scientific, Whaltham, MA, USA), according to the manufacturer’s instructions. Each sample was tagged with a different TMT tag, and samples were analyzed in two different multiplex experiments. In order to label the samples, the peptide mixture was incubated with the prepared TMT reagent for 2 h, at room temperature. Then, samples were pooled in the two different experiments, described here:

(i) Multiplex experiment 1 contained nine samples: FD1, FD2, AR1, AR2, AR-AO1, AR-AO2, FD5, AR3, and AR-AO5. These samples were respectively tagged with the following TMT tags: 127N, 127C, 128N, 128C, 129N, 129C, 130N, 130C, and 131. The experiment also included an internal control with the TMT tag 126.

(ii) Multiplex experiment 2 contained six samples: FD4, AR4, AR-AO4, FD3, AR5, and AR-AO3. These samples were respectively tagged with the following TMT tags: 127C, 128C, 129C, 130N, 130C, and 131. The experiment also included an internal control with the TMT tag 126.

Pooled samples were dried and reconstituted in 0.1% formic acid. Finally, peptide clean up and fractionation was performed using Pierce C18 spin columns (Thermo Fisher Scientific, Whaltham, MA, USA) following manufacturer’s protocol.

#### 2.2.3. Liquid Chromatography and Mass Spectrometry Analysis

The TMT-labeled tryptic peptide mixture, and peptide clean up and fractionation, were performed using reverse-phase nanoscale liquid chromatography (nanoLC) with a trap column C-18 (L 2 cm, 100 μm ID, 5 μm, Thermo Fisher Scientific, Whaltham, MA, USA) and an analytical column (L 15 cm, 75 μm ID, 3 μm, Thermo Fisher Scientific, Whaltham, MA, USA), and with a 3–97% acetonitrile gradient, in 0.1% acid formic for 125 min at a flow rate of 350 nL/min. Subsequently, an LTQ Orbitrap Velos (Thermo Fisher Scientific, Whaltham, MA, USA) was used to obtain MS/MS spectra for each peptide.

#### 2.2.4. Protein Identification, Quantification, and Data Analysis

All protein-based raw data were searched against the Swiss-Prot in-house protein FASTA file with *Homo sapiens* sequences, a total of 23733 proteins, using SEQUEST HT version 28.0 (Thermo Fisher Scientific, Whaltham, MA, USA). The following settings were used for searches: two missing cleavages for trypsin, 0.6 Da fragment mass tolerance, 20 ppm precursor mass tolerance, with carbamidomethylation on Cys specified as the fixed modification, and oxidation on Met specified as variable modification.

The criteria to accept protein identification was adjusted to 1% of false discovery rate (FDR) and at least one unique peptide per protein. The normalized TMT quantification of relative protein values was calculated as the ratio between the reported ion intensities from each individual sample and the reported ion intensity from the internal control. To minimize the sample-derived contamination effect of the cryopreservation medium, the spectra identified as chicken-derived proteins were excluded from the normalization analysis. The differentially expressed protein (DEP) cutoff value was set at over a 1.5-fold change for upregulated proteins and a 0.67-fold change for downregulated proteins, with *p* < 0.05 calculated by Student’s *t*-test.

#### 2.2.5. Functional Enrichment Analysis

The determination of the percentage of proteins belonging to “molecular function”, “biological process”, and “cellular component” categories was performed using the PANTHER database (Protein Analysis Through Evolutionary Relationships, http://pantherdb.org; accessed on 15 January 2020); the obtained data were incorporated into a graph.

Protein–protein interaction networks were identified using the STRING database (version 8.3; String Consortium; http://string.embl.de/, accessed on 15 January 2020) to establish the interactions of identified proteins with a specific group of molecules.

## 3. Results

### 3.1. Sperm DNA Fragmentation

All samples were tested for their DNA fragmentation values: DSB DNA damage using the neutral comet assay, and oxidative DNA damage using the alkaline comet assay. Results are shown in Table 1. The alkaline comet assay did not show differences among FD, RM, and RM-OA groups (*p* > 0.05). However, the neutral comet assay showed statistically significant differences between FD and RM (*p* = 0.008), but not between FD and RM-OA (*p* = 0.111), nor between RM and RM-OA (*p* = 0.062).

### 3.2. Analysis of Differentially Expressed Proteins (DEPs)

TMT labeling following LC–MS/MS analysis resulted in the identification of 607 proteins with unique peptides or polypeptide segments. In comparison with fertile donors, the RM group showed 24 proteins significantly upregulated, and six significantly downregulated. Similarly, the RM group after oral antioxidant treatment showed 25 proteins upregulated, and five downregulated, when compared to fertile donors (Table 2). Greater changes in protein levels were detected for the PR domain zinc finger protein 2 (PRDM2, 4.49-fold, *p* < 0.01), microtubule-associated protein 2 (MAP2, 3.20-fold, *p* < 0.01), A-kinase anchor protein 4 (AKAP4, 2.49-fold, *p* < 0.01), and zinc finger protein basonuclin-2 (BNC2, 0.36-fold each, *p* < 0.01). Although no DEPs were found between RM and RM-AO, we were able to identify proteins that showed significant differential expression (*p* < 0.05) between RM and FD, but not between RM-AO and FD (*p* > 0.05), indirectly indicating an improvement after antioxidant treatment. The proteins with improved expression after the treatment were: LIM domain only protein 7 (LMO7), matrix metalloproteinase-28 (MMP28), neurofilament heavy polypeptide (NEFH), and BNC2. Proteins that also improved their expression level, although to a minor extent, were: PRDM2, ankyrin repeat and SOCS box protein 14 (ASB14), calcium-binding tyrosine phosphorylation-regulated protein (CABYR), chromosome-associated kinesin KIF4B, MAP2, and sperm surface protein Sp17.

### 3.3. Functional Enrichment and Clustering Analyses of DEPs

**Figure 1:**
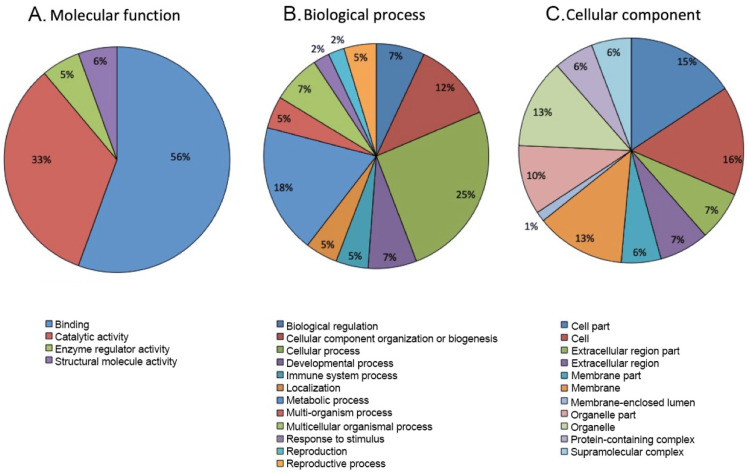
Classification and annotation of DEPs by PANTHER database (Protein Analysis Through Evolutionary Relationships). GO analysis of DEPs in the sperm of recurrent miscarriage patients before and after antioxidant treatment vs. fertile donors. Proteins were classified by their molecular function (**A**), biological process (**B**), and cellular component (**C**).

Functional enrichment analyses were performed to investigate the biological functions, processes, and cellular localization of DEPs. Classification was based on gene ontology (GO) using the PANTHER 15.0 bioinformatics software platform. DEPs in both RM and RM-OA groups had similar GO enrichment classification. The analysis revealed that DEPs were largely involved in binding and catalytic activity; other functions were enzyme regulator activity and structural molecule activity as shown in (Figure 1A). As for biological processes, about 40% of DEPs were found to be related to cellular and metabolic processes and, less frequently to component organization or biogenesis (Figure 1B). Developmental process, reproduction and reproductive processes added up to 13.95% of the DEPs. Finally, DEPs were found to belong to the cell (16%) and cell component (15%), to organelle or organelle part (23%) and membrane (13%) (Figure 1C).

**Figure 2:**
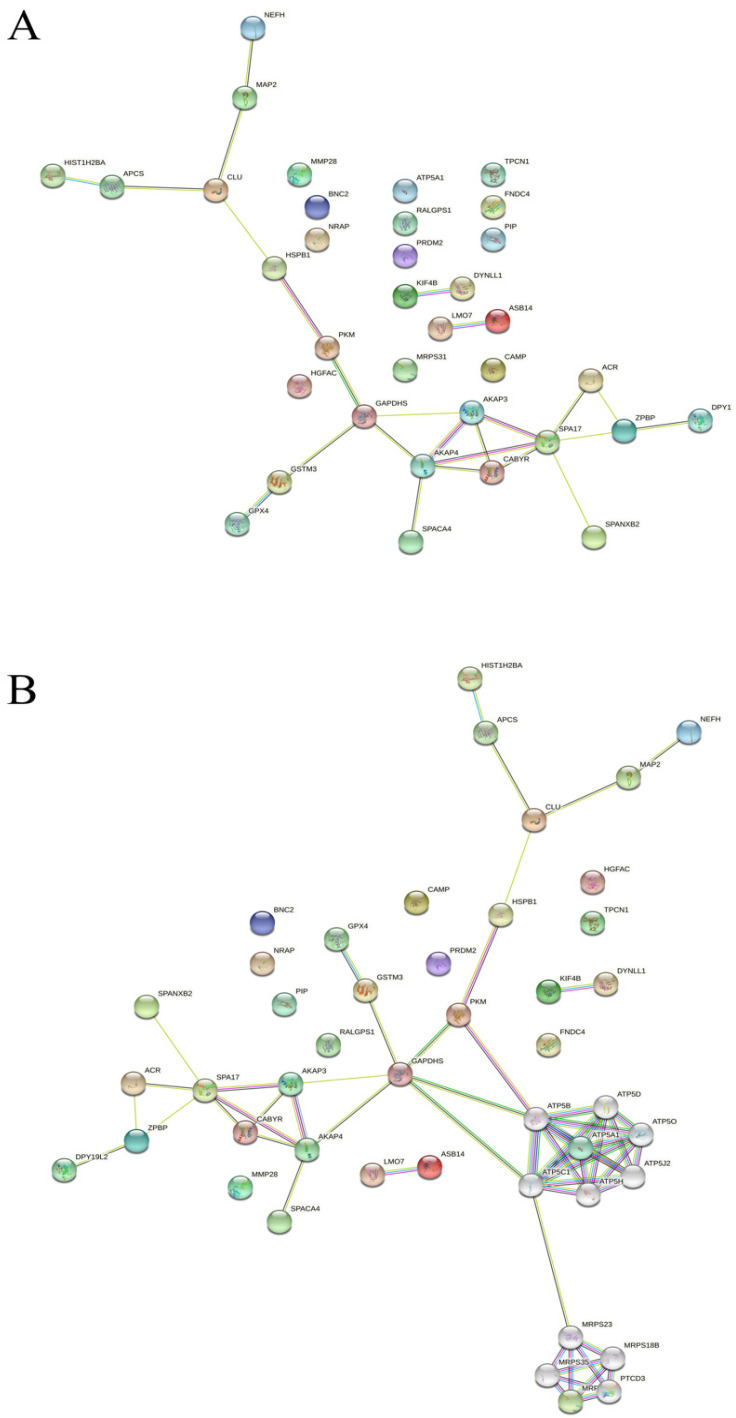
Map of protein–protein interactions of DEPs analyzed by Search Tool for the Retrieval of Interacting Genes/Proteins (STRING) in first shell, direct interconnections (**A**) and second shell, indirect interconnections (**B**).

To create protein–protein interaction networks for identified DEPs in the sperm of RM and RM-OA patients, and FDs, STRING 11.0 software was used. The generated network shows proteins as nodes and interactions as colored lines between them. Our search revealed that most proteins were clustered into nodes related to: cytoskeleton (mainly related to sperm surface), metabolism, and DNA–histone interaction (Figure 2A). Moreover, in a second level of association, most of the clustered proteins were involved in the ATP synthase complex and were mitochondrial ribosomal proteins; all nodes of interaction were connected by PKD (Figure 2B).

## 4. Discussion

The search for sperm protein biomarkers is crucial in the human reproduction field. It is believed that, when found, these biomarkers will allow researchers and clinicians to better characterize recurrent miscarriage infertile couples. Moreover, they will help establish a new generation of personalized and directed therapies. In the case of idiopathic RM patients, it has been proposed that the increased presence of sperm with DNA DSBs is a male condition that leads to a higher risk when couples do not present female factors [17]. Despite this, we found that fertile donors may also present increased incidence of sperm with increased DSB occurrence. Thus, identifying DEPs between FDs and RM patients with increased DSB occurrence may provide some insights into the pathophysiology of RM. Here, we tested the proteomic differences between RM patients with sperm DNA DSB damage and fertile donors, and the effect of antioxidants on these patients.

Comparative proteomics studies have previously been conducted on sperm [29,30,31]. However, RM patients have only been included in a few recent reports [23,32]. To our knowledge, this is the first study that analyzes the protein profile of patients categorized as being at high risk of RM according to their sperm DNA DSB profile. In addition, we assessed these patients both before and after oral antioxidant treatment, and compared their profiles with the fertile donors’ proteomic profiles to elucidate whether the treatment changed the protein expression profile. The use of TMT in quantitative proteomics permits the analysis of multiple groups within the same experimental design with high resolution. Here, a total of 607 proteins from 15 semen samples were identified and quantified. A total of 33 proteins showed differential expression between fertile donors and RM and RM-OA groups. Some of them were previously described in RM and in patients with high incidence of reactive oxygen species, including AKAP4, histone H2B type 1-A (H2B1A), prolactin-induced protein (PIP), glyceraldehyde-3-phosphate dehydrogenase (G3PT), sperm protein associated with the nucleus X chromosome (SPANXB), and clusterin (CLU), among others [23,32,33]. However, other DEPs are described here for the first time in the RM group, such as PRDM2, serum amyloid P component (SAMP), LMO7, and probable C-mannosyltransferase DPY19L2.

No differences were found regarding sperm DNA DSBs between RM patients and RM-OA patients, a fact that indicates that the DSBs generation pathway is not related to oxidative stress, as suggested previously [34]. Similarly, no significant differences in protein abundance were directly found between these two groups. Due to this finding, we focused on the proteins that, after antioxidant treatment, shifted their expression levels towards the expression levels found in FDs, thus providing indirect evidence of protein expression improvement after antioxidant treatment. This indirect measurement showed shifts in the expression of LMO7, NEFH, and BNC2 in the RM-OA group towards the expression levels found in FDs. The expression levels of these proteins in the RM-OA group turned out to be similar to the expression levels observed in the FD group, suggesting that they could be somehow related to the physiopathology of the RM patients with increased DSB occurrence and oxidative stress. To the best of our knowledge, the present study is the first to conduct a proteomic analysis on the effects of an antioxidant treatment on sperm from recurrent miscarriage couples, and the proteins identified should be further validated in large cohorts of patients.

GO enrichment analysis revealed that functions of the identified proteins in RM and RM-OA groups were very similar, and were significantly enriched in binding and catalytic functions and in cellular and metabolic processes, suggesting that these pathways are important to the etiology of RM infertility. These results are in accordance with previous studies [23,32]. Additionally, more than 15% of DEPs were involved in developmental or reproductive functions, indicating a relationship with sperm fertility. Finally, as expected, the analysis of protein localization showed that DEPs derived mostly from the cell and organelles, not from the extracellular fluid. Membrane proteins accounted for 15.6%; these included AKAP3 and AKAP4, MAP2, CABYR, probable C-mannosyltransferase DPY19L2, zona pellucida binding protein 1 (ZP1), and two-pore calcium channel protein 1 (TPC1). These could be interesting as new protein biomarkers for sperm selection before an ART treatment. Despite the fact that DSB assessment is useful as a diagnostic biomarker, it cannot be used for this purpose, as cells need to be lysed for analysis.

Protein–protein interactions among DEPs showed clusters in network edges (Figure 2A) related to: (1) cellular metabolism, (2) cytoskeleton (motility and sperm–egg fusion), and (3) DNA damage. The following four proteins downregulated in RM samples were directly involved in glycolysis and oxide reduction processes: pyruvate kinase PKM (KPYM), glyceraldehyde-3-phosphate dehydrogenase (G3PT), phospholipid hydroperoxide glutathione peroxidase (GPX4), and mitochondrial membrane ATP synthase (ATPA). Most of these proteins have a demonstrated sensibility to ROS, and their function is related to maintaining the cellular redox status [35]. However, after antioxidant treatment, G3PT, GPX4, and ATPA did not shift towards FD levels, suggesting that the oxidant–antioxidant status should be assessed, taking into account both sperm and seminal plasma [20,25]. Interestingly, the protein GPX4 is an essential antioxidant enzyme that protects cells from ROS [36], and is localized in three different cell components: the cytosol, mitochondria, and nucleus. In the nucleus, GPX4 is associated with matrix attachment regions (MAR), and has a role in DNA condensation during spermiogenesis [37], as well as in the paternal chromatin decondensation that follows fertilization, with a possible impact on embryo kinetics and development [38,39]. Similarly, DSBs have also been described to be localized in the MAR regions, possibly to facilitate their repair during the first steps of embryogenesis [40,41]. Therefore, the colocalization of both GPX4 and DSBs may indicate that this protein could be somehow responsible for DSB repair in early embryogenesis.

The cytoskeleton is very important to sperm cells since it is responsible for cell movement and shape, as well as sperm–egg fusion, acrosome reaction, and vesicular transport. Our results show a downregulation of nebulin-related anchoring protein in RM and RM-AO, and an upregulation of AKAP3, AKAP4, MAP2, CABYR, MMP-28, probable C-mannosyltransferase DPY19L2, Ras-specific guanine nucleotide-releasing factor RalGPS1, and sperm surface protein Sp17. Interestingly, neurofilament heavy polypeptide (NEFH) improved its expression after treatment with oral antioxidants, resulting in nonsignificant differences between FD and RM-AO. To our knowledge, this is the first report of NEFH in human sperm. NEFH has been previously characterized in the subacrosomal zone of shrimp sperm, along with MAP2 and tau proteins [42]. It has been shown that MAP2 is expressed in human sperm [43], where it interacts with neurofilaments and microtubules to stabilize their growth [44].

Protein regulation, such as folding, and post-translational modifications, such as ubiquitination, phosphorylation, etc., are very important for the maintenance of cellular processes. Our analysis shows that two proteins involved in protein regulation, ASB14 and heat shock protein beta-1 (HSPB1), were upregulated in FD vs. RM and RM-OA groups. LMO7 was also upregulated, but only in the RM group. In the RM-OA group, LMO7 improved its expression profile, resulting in nonsignificant differences compared to the FD group. This protein is implicated in important biological functions, possibly through a suspected role in the ubiquitin-protein transferase process and actinin binding. PPI revealed its interaction with ASB14, which is an ankyrin repeat and cytokine signaling (SOCS) box (ASB) family protein that regulates proteins via polyubiquitination [45]. LMO7 functions have been related to embryonic cell death and to actin cytoskeleton organization via cell–cell junctions or cell–matrix junctions during embryonic development and oncogenesis [46,47]. The LIM domain of LMO7 has also been associated with the control of mitosis progression, and with exerting an effect on SAC (spindle assembly checkpoint). HSPB1, on the other hand, is a protein regulated by cellular stressors such as ROS. It participates in the cell cytoskeleton maintenance [48], and its presence in high concentrations in sperm has been related to poor blastocyst quality. CLU is another protein that has also been related to poor blastocyst quality [49]. CLU also protects against ROS, participating in multiple biological processes such as stress response, cell cycle control, and DNA repair in apoptosis [32,50]. Recently, CLU has been proposed as a new biomarker due to its role as a paternal factor regulator [51]; however, in our study, we did not observe a change in its expression after antioxidant treatment.

Our analysis also found DEPs with a nuclear location that could be involved in DNA damage, including chromosome-associated kinesin KIF4B, H2B1A, PRDM2, serum amyloid P component (SAP), sperm protein associated with the nucleus on the X chromosome B1 (SPANXB1), and BNC2. Sperm DNA is initially packaged by histones that are subsequently replaced by protamines during spermiogenesis. It has been demonstrated that alterations in this replacement process cause defects in chromatin packaging and damage to sperm DNA [52,53,54]. An increased presence of H2B1A in the sperm nucleus has been linked to decreased mobility, DNA compaction anomalies, and increased sperm DNA fragmentation [55]. It has been suggested that PRDM2 is a component of the double-strand break repair complex. This component is critical for maintaining DNA integrity [56], and dysregulation of PRDM2 concentration in the sperm nucleus might be involved in the origin of sperm DNA fragmentation. Regarding SAP, it had previously been described in samples from infertile patients [57,58], and it had also been shown that apoptotic cells carry chromatin fragments coated with SAP on their surface [59]. In a previous study, SAP correlated with infertile patients who showed high sperm DNA fragmentation and poor embryonic quality [60]. Finally, BNC2 improved its profile in patients after antioxidant treatment, and was found to be underexpressed. This protein has been described as a DNA-binding protein that regulates meiosis and mitosis in male germ cells, and a previous study has shown that it is involved in the activation of the paternal genome after fertilization [61].

### Limitations

Our study was conducted on a very specific group of couples, with recurrent miscarriage and double-strand breaks, and most of the female factors were excluded. Moreover, although we assessed the samples in parallel before and after an antioxidant treatment, adding robustness to the analysis, the sample size of the biological subjects was limited (*n* = 5). With this sample size, post hoc calculation of the statistical power following the method of Dhand and Khatkar (2014) showed that our study presents a power of 80% when a fold change is >2 for upregulated proteins or <0.5 for downregulated proteins. This indicates that although the proteomic analysis is robust and all upregulated and downregulated proteins are reliable within the patients analyzed, the proteins with a fold change within the ranges described above may represent more important targets to be validated in larger cohorts; thus, one should focus on these proteins to expand the research in the biomarkers for RM. Such validation should also take into account the presence of the same proteins in seminal plasma, especially when proteins are related to the antioxidant family. Finally, as stated in the above discussion, no direct differences were found between RM and RM-OA, and differentially expressed proteins were described according to the proteomic profile found in fertile donors, which is the other limitation of our analysis.

## 5. Conclusions

In conclusion, the present study analyzed for the first time, the DEP profiles of RM patients with high DSB occurrences before and after oral antioxidant treatment. No significant differences between the RM and RM-OA group were directly found, but some proteins were found to change their expression after treatment, achieving a profile closer to fertile donor values. This was the case for LMO7, NEFH, and BNC2. In addition, the DEPs detected in this study between fertile donors and RM groups were vulnerable to oxidative stress, and were implicated in cytoskeleton organization, acrosome reaction, and ATP production for sperm motility. H2B1A, GPX4, and PRM2 proteins were found to be deregulated, and may be involved in the sperm DNA DSBs that characterized our RM cohort through their role in DNA remodeling and repair, and paternal DNA decondensation in the embryo. Further studies should analyze the specific effect of each protein on embryo development to improve the knowledge of the molecular basis of RM disease.

## Figures and Tables

**Table 1:** Sperm DNA fragmentation (mean ± SD) outputs for fertile donors and unexplained RM patients before and after oral antioxidant treatment.

Group and Pathology	Oxidative DNA Damage (Alkaline Comet)	Double-Strand Breaks (Neutral Comet)
FD (*n* = 5)	37.4 ± 12.64	51.6 ± 14.18
RM (*n* = 5)	43.8 ± 11.07	75.6 ± 4.04 *
RM-OA (*n* = 5)	43.6 ± 1.51	64.2 ± 6.09

* Statistically significant differences compared to FD (*p* < 0.05).

**Table 2:** Differentially expressed proteins in human spermatozoa from fertile donors (FD) vs. recurrent miscarriage patients before (RM) and after (RM-OA) oral antioxidant treatment. * Indicates proteins that are NOT different in one of the two comparisons.

			FD vs. RM		FD vs. RM-OA	
Protein Name	Gene Name	Uniprot Ac	Ratio FD/RM	*p*-Value	Ratio FD/RM-OA	*p*-Value
Upregulated						
28S ribosomal protein S31, mitochondrial	RT31_HUMAN	Q92665	1.86	0.037	1.61	0.037
Acrosin	ACRO_HUMAN	P10323	2.03	0.002	1.89	0.001
A-kinase anchor protein 3	AKAP3_HUMAN	O75969	1.69	0.005	1.70	0.007
A-kinase anchor protein 4	AKAP4_HUMAN	Q5JQC9	2.49	0.023	2.58	0.014
Ankyrin repeat and SOCS box protein 14	ASB14_HUMAN	A6NK59	2.53	0.011	2.09	0.015
ATP synthase subunit alpha, mitochondrial	ATPA_HUMAN	P25705	1.49	0.018	1.63	0.008
Calcium-binding tyrosine phosphorylation-regulated protein	CABYR_HUMAN	O75952	2.10	0.001	1.59	0.005
Cathelicidin antimicrobial peptide	CAMP_HUMAN	P49913	1.56	0.045	1.69	0.022
Chromosome-associated kinesin KI4B	KIF4B_HUMAN	Q2VIQ3	2.10	0.001	1.59	0.006
Dynein light chain 1, cytoplasmic	DYL1_HUMAN	P63167	1.89	0.060*	2.20	0.020
Glyceraldehyde-3-phosphate dehydrogenase, testis-specific	G3PT_HUMAN	O14556	1.92	0.015	1.98	0.010
Histone H2B type 1-A	H2B1A_HUMAN	Q96A08	2.17	0.002	2.33	<0.001
Heat shock protein beta-1	HSPB1_HUMAN	P04792	2.30	0.012	2.67	0.003
LIM domain only protein 7	LMO7_HUMAN	Q8WWI1	1.63	0.026	1.12	0.632 *
Matrix metalloproteinase-28	MMP28_HUMAN	Q9H239	1.99	0.008	1.64	0.033
Microtubule-associated protein 2	MTAP2_HUMAN	P11137	3.20	<0.001	2.54	<0.001
Neurofilament heavy polypeptide	NEFH_HUMAN	P12036	1.72	0.028	1.49	0.056 *
Phospholipid hydroperoxide glutathione peroxidase	GPX4_HUMAN	P36969	1.80	0.024	2.08	0.002
PR domain zinc finger protein 2	PRDM2_HUMAN	Q13029	4.49	0.012	2.76	0.022
Probable C-mannosyltransferase DPY19L2	D19L2_HUMAN	Q6NUT2	2.19	0.019	2.05	0.022
Pyruvate kinase PKM	KPYM_HUMAN	P14618	2.03	0.009	1.85	0.010
Serum amyloid P component	SAMP_HUMAN	P02743	1.96	0.011	1.76	0.049
Sperm acrosome membrane-associated protein 4	SACA4_HUMAN	Q8TDM5	1.61	0.067 *	1.86	0.027
Sperm protein associated with the nucleus on the X chromosome B1	SPNXB_HUMAN	Q9NS25	1.56	0.030	2.14	0.001
Sperm surface protein Sp17	SP17_HUMAN	Q15506	2.73	0.001	2.19	0.001
Two-pore calcium channel protein 1	TPC1_HUMAN	Q9ULQ1	1.74	0.007	1.60	0.029
Zona pellucida binding protein 1	ZPBP1_HUMAN	Q9BS86	1.84	0.065*	1.96	0.018
Downregulated						
Clusterin	CLUS_HUMAN	P10909	0.47	<0.001	0.52	0.001
Hepatocyte growth factor activator	HGFA_HUMAN	Q04756	0.60	0.019	0.48	<0.001
Nebulin-related anchoring protein	NRAP_HUMAN	Q86VF7	0.37	0.024	0.38	0.014
Prolactin-inducible protein	PIP_HUMAN	P12273	0.44	0.006	0.54	<0.001
Ras-specific guanine nucleotide-releasing factor RalGPS1	RGPS1_HUMAN	Q5JS13	0.29	0.012	0.37	0.001
Zinc finger protein basonuclin-2	BNC2_HUMAN	Q6ZN30	0.36	0.046	0.43	0.065 *

* Proteins that did not show statistical differences with the specific group compared.

## Data Availability

Data generated during the current study are available from the corresponding author on reasonable request.

## Abbreviations

DSB	Double-stranded breaks
DEP	Differentially expressed protein
FD	Fertile donors
GO	Gene ontology
LC	Liquid chromatography
MS	Mass spectrometry
RM	Recurrent miscarriage
RM-OA	Recurrent miscarriage after oral antioxidant treatment
ROS	Reactive oxygen species
SDF	Sperm DNA fragmentation
TMT	Tandem mass tag
